# Similarities in the Age-Specific Incidence of Colon and Testicular Cancers

**DOI:** 10.1371/journal.pone.0066694

**Published:** 2013-06-26

**Authors:** Luis Soto-Ortiz, James P. Brody

**Affiliations:** Department of Biomedical Engineering, University of California Irvine, Irvine, California, United States of America; Queen's University Belfast, United Kingdom

## Abstract

Colon cancers are thought to be an inevitable result of aging, while testicular cancers are thought to develop in only a small fraction of men, beginning *in utero*. These models of carcinogenesis are, in part, based upon age-specific incidence data. The specific incidence for colon cancer appears to monotonically increase with age, while that of testicular cancer increases to a maximum value at about 35 years of age, then declines to nearly zero by the age of 80. We hypothesized that the age-specific incidence for these two cancers is similar; the apparent difference is caused by a longer development time for colon cancer and the lack of age-specific incidence data for people over 84 years of age. Here we show that a single distribution can describe the age-specific incidence of both colon carcinoma and testicular cancer. Furthermore, this distribution predicts that the specific incidence of colon cancer should reach a maximum at about age 90 and then decrease. Data on the incidence of colon carcinoma for women aged 85–99, acquired from SEER and the US Census, is consistent with this prediction. We conclude that the age specific data for testicular cancers and colon cancers is similar, suggesting that the underlying process leading to the development of these two forms of cancer may be similar.

## Introduction

It is widely thought that colon carcinoma is an inevitable result of aging [1]–[4]. A colon tumor develops when a cell accumulates a sufficient set of mutations [5], [6]. These mutations accumulate throughout life. Thus, the longer one lives, the more likely one is to develop a tumor in the colon.

On the other hand, it is thought that the propensity to develop a testicular germ cell tumor exists early in life and in only a small sub population [7]. The incidence of one of the most common forms of testicular cancer, germ cell seminoma, peaks in the early 30's and decreases to near zero by the age of 60 [8].

Colon and testicular cancers, like most forms of solid cancer, share some common characteristics. They each start from a single cell. Initially, this cell is no different than the many similar cells that exist in each tissue. A tumor develops, and is diagnosed, when one of these cells accumulates a sufficient set of mutations.

Statistical models of the age-specific incidence provide information on the carcinogenesis process [9]–[11]. The textbook model of the age-specific incidence data was developed by Armitage and Doll in 1954 [12]–[14]. In this model, the incidence, 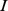, increases with age, 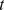, as a power law, 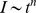, where 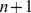 is the number of rate-limiting steps, often interpreted as the number of mutations. This model led to the widely cited notion that most cancers are caused by four to six mutations [5].

The age-specific incidence data has improved significantly since 1954. In 1954, the data was based upon mortality and collected from death certificates. The SEER network of cancer registries began in 1972 with nine different cancer registries. These registries compile standardized information on all cancers diagnosed within specific geographic areas. As the SEER data became available, it became clear that the age-specific incidence data deviates from the Armitage Doll model [13].

To account for the difference between the age-specific incidence and the Armitage Doll model, Moolgavkar, Venzon, and Knudson developed the two-stage with clonal evolution model in 1979 [15]–[18]. This model postulated that a cell needed two rate limiting steps, followed by clonal expansion. This model provided a better fit to the age-specific incidence data, especially for colon cancer for ages less than 85 years of age.

**Figure 1:**
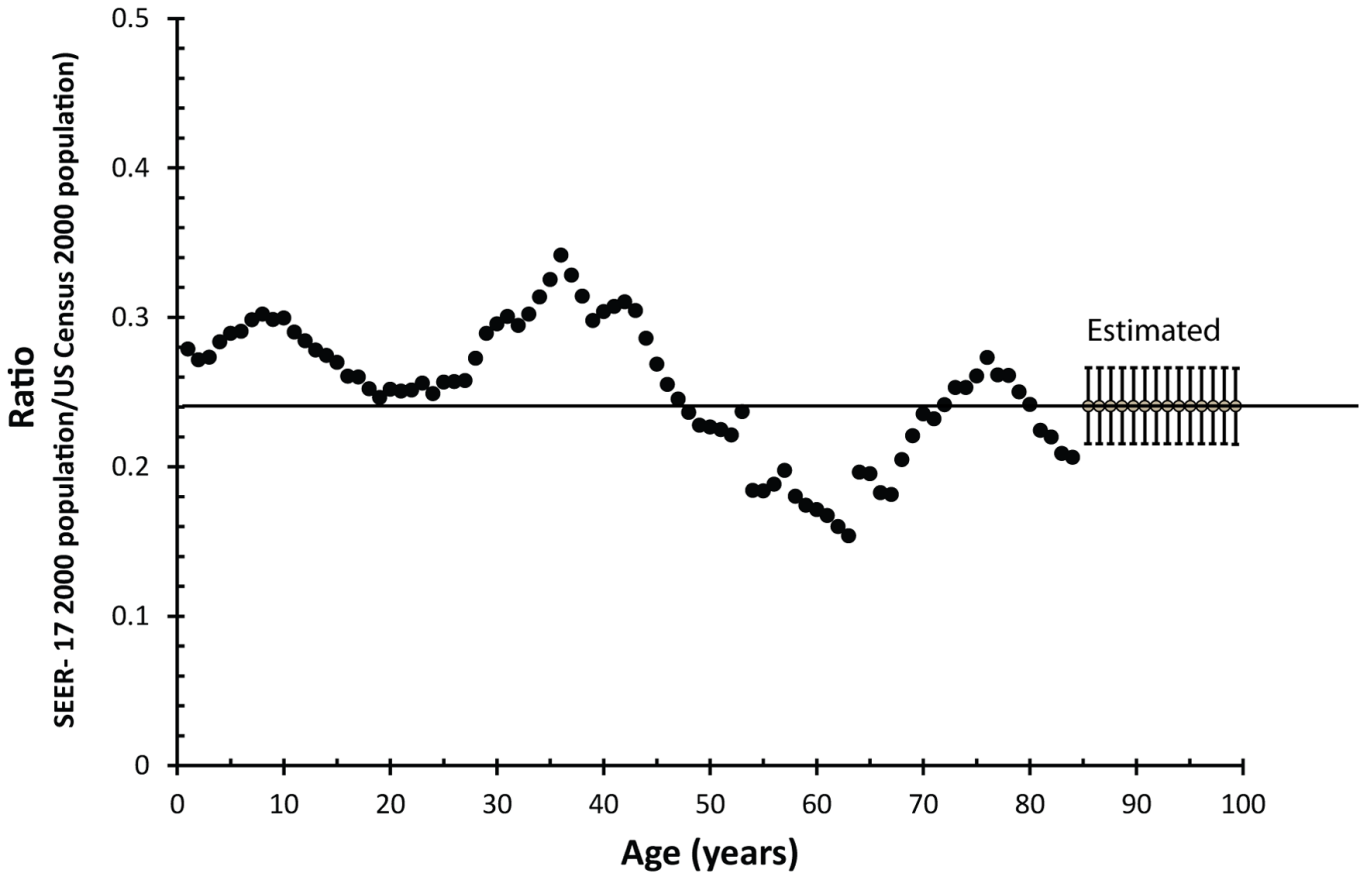
The ratio of the 2000 SEER-17 population to the 2000 US Census population is plotted as a function of age. To estimate the population by age of 85 to 99 year old people, we found the best match between the US population and the SEER-17 population for ages 60–84, indicated by the solid line at 0.24. We then used that multiplied that ratio (0.24) by the measured 2000 US Census population by age for 85 to 99 year olds, to obtain an estimate of the SEER-17 population by age for ages 85–99. This figure shows the estimates for ages 85–99, along with associated error and the ratio for ages 0–84 years of age.

The statistical power of the age-specific incidence data continued to increase over time. SEER now contains 18 geographically distinct registries containing over a quarter of the US population, see Figure 1.

With the modern SEER data, it became clear that the specific incidence of colon carcinoma flattens or even decreases with age after about 80 years [18]–[24]. To account for this decrease, Pompei and Wilson developed an ad-hoc beta model [20] of the age-specific incidence. This model postulated that an age-dependent process exists where cell division slows. This process leads to a decrease in the cancer incidence rate at advanced ages. The mathematical model is formulated with a mathematical beta function, thus its name.

Tumors are diagnosed when the first of many similar cells accumulate a sufficient set of mutations. All three of these models (Armitage Doll/ two stage with clonal expansion/ beta distribution) provide a mathematical formula that estimates the probability of a cell developing into a tumor. The hazard function of the probability distribution is associated with the age-specific incidence data.

In contrast to the beta model, we have proposed that the age-specific incidence data should follow an extreme value distribution. The extreme value distribution model of the age-specific incidence is based upon three well established assumptions [25]:

1. Cancer starts in a single cell [26].
2. This single cell is not special, many similar cells exist in a tissue.
3. A tumor is diagnosed when the first of these many similar cells has accumulated a sufficient set of mutations.

One characteristic of the extreme value model is that it neither depends upon the details of how a cell acquires mutations nor the rate at which the cell proliferates.

In this paper, we test the hypothesis that the age-specific incidence for testicular cancer seminomas and for colon carcinoma follow the same distribution. Testicular cancer is typically diagnosed in 20–40 year old men, while colon carcinoma is usually diagnosed in people over 50 years of age.

## Results

**Figure 2:**
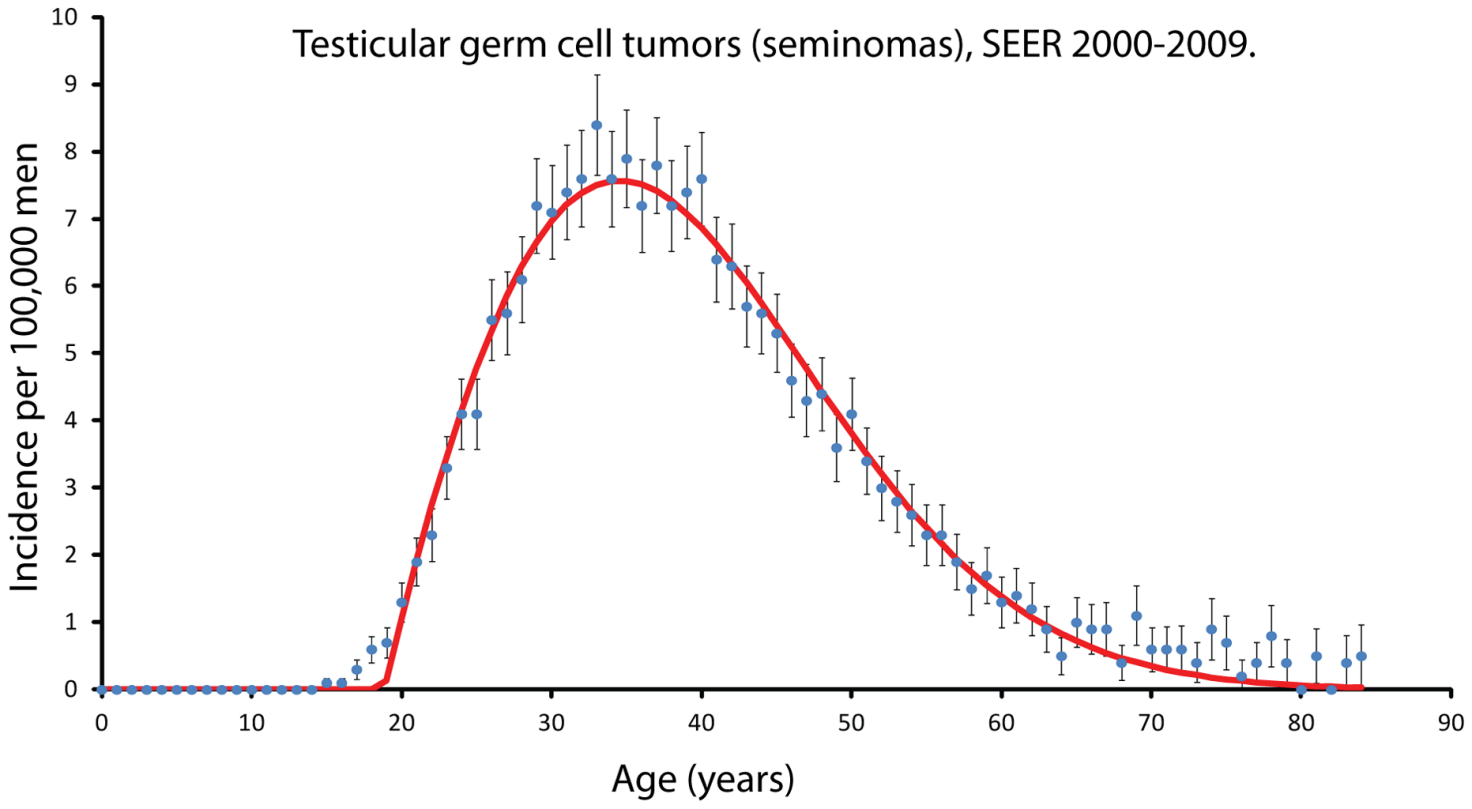
The specific incidence, as recorded by the SEER-17 cancer registries from 2000–2009, for testicular germ cell tumors (seminomas) is shown as a function of age. The points indicate the measured values and the error bars indicate 95 % confidence intervals. The incidence reaches a maximum in the early 30's and decreases to near zero. The solid line indicates the best fit Weibull distribution. The agreement between the data and the line supports the extreme value model. The area under the line is about 207, which indicates that in a population of 100000 men only about 207 men are susceptible to developing this form of cancer. The actual number of tumors that develop in those 100000 men will be lower than 207 because some men will die before they develop testicular cancer.

The age-specific incidence of testicular cancer is consistent with the Weibull distribution. The parameters of the best fit Weibull distribution are 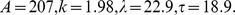 The best fit Weibull distribution is shown in Figure 2. It has a 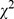 value of 66 with 42 degrees of freedom (ages 20–67). The data diverges from the predicted distribution at ages 17–20. This difference may be due to heterogeneous aging of the population.

The age-specific incidence of colon carcinoma is also consistent with the Weibull model, for ages 30–84. The model does not account for some artifacts in the data, contributing to the high 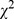 value. Two noticeable artifacts are an increase at ages 50 and 51, probably due to screening for colorectal cancers, and the increase at age 65, probably due to access to universal medical care.

The data cannot clearly distinguish between a model in which 100% of the population is susceptible (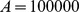) and one in which a small percentage of the population is susceptible (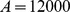).

**Figure 3:**
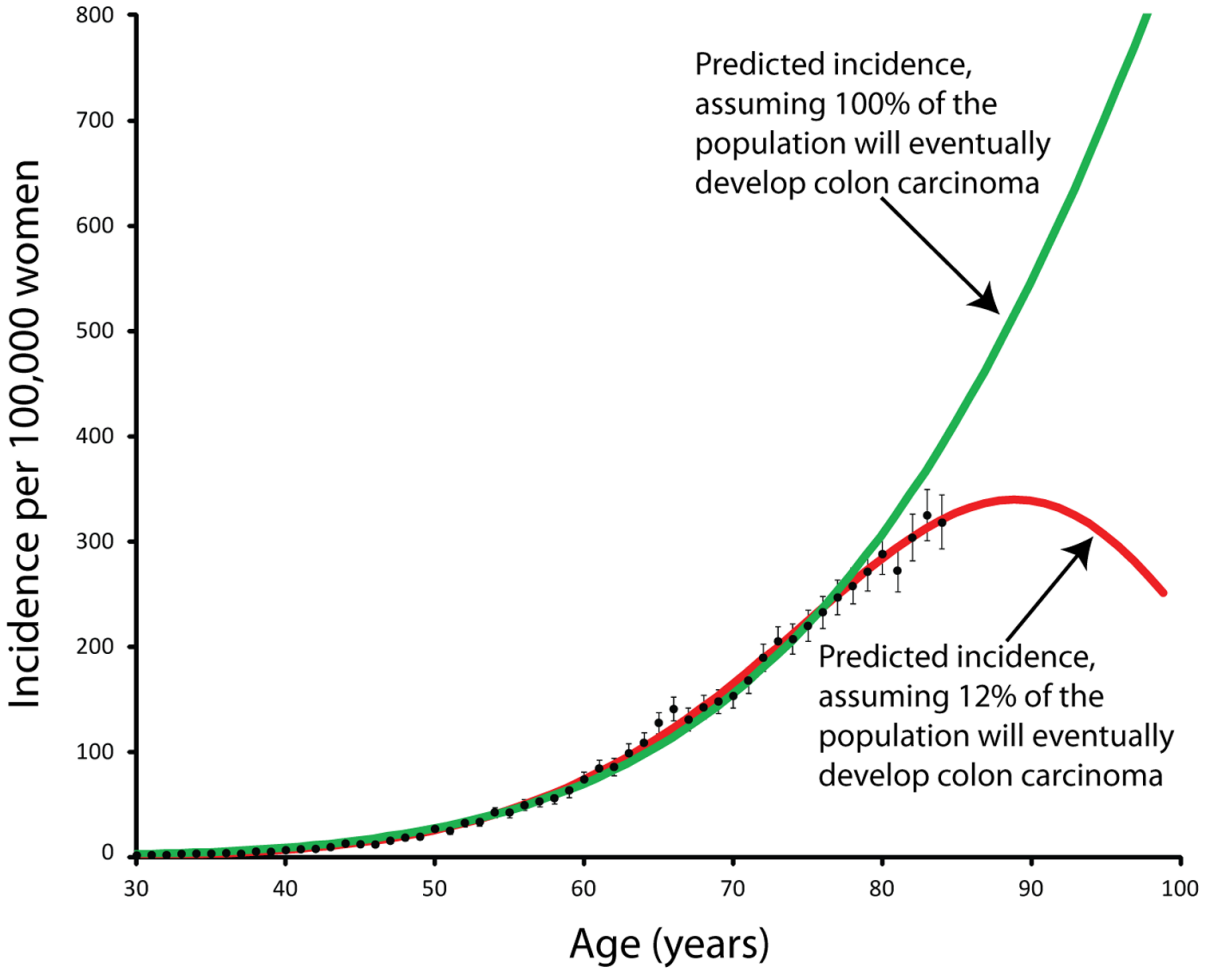
This figure presents the incidence of colon cancer during the year 2000 as measured by the SEER-17 cancer registries. Two fitted Weibull distributions are shown. The first assumes that 100% of the population will eventually develop colon carcinoma, while the second makes no assumption about the fraction of the population that will develop colon carcinoma, but instead fits it as a parameter. The best fit parameter is about 12% of the population, while the line representing 100% of the population is a good fit, but not perfect. More data in the 85–99 range will clearly delineate between the two cases.

The green line in Figure 3 represents the best fit Weibull distribution to the data when two parameters are allowed to vary (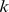 and 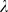, 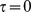) while 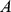 was fixed to be 100000, or 100% of the population. This provides an acceptable fit, as characterized by a 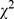 value of 252 with 54 degrees of freedom when 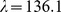 and 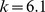.

The red line in Figure 3 represents the best fit Weibull distribution to the data when three parameters are allowed to vary (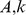 and 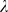; 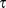 was fixed to be zero after preliminary analysis showed that the best fit value was consistent with zero.) The 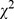 value was minimized when 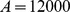, 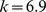, and 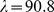. This provided an excellent fit to the data, as characterized by 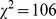 with 53 degrees of freedom.

**Figure 4:**
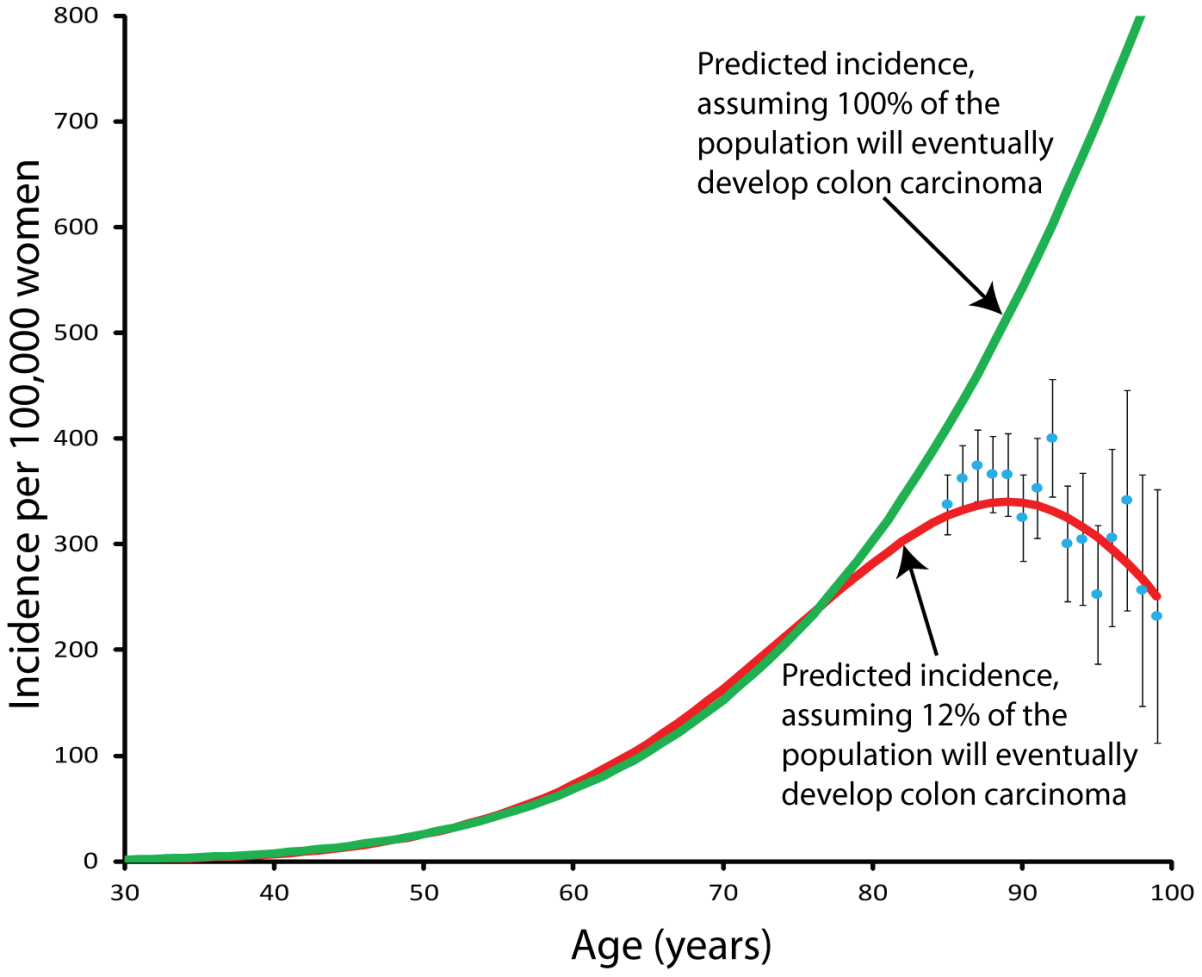
This graph presents the data for colon carcinoma among women in 2000 as collected by the SEER-17 cancer registries for the ages 85–99. Two lines are shown, one represents the assumption of 100% of the population, the second represents the best fit data from the 0 to 84 years old data, with 12% susceptible. The data points indicate the measured specific incidence for women 85–99 years old. The data points clearly fall upon the 12% line, ruling out the 100% hypothesis.

The data for 0–84 years of age do not conclusively rule out either model. To distinguish between the two models, we compiled measurements for the specific incidence of colon carcinoma from 85 to 99 years of age, as shown by the blue circles in Figure 4. With this data, the hypothesis that 100% of the population is susceptible to colon carcinoma can be ruled out.

## Discussion

Cancer is widely thought to be a disease of aging [27]–[29]. This assertion has been used to support the hypothesis that tumor-suppressor mechanisms contribute to aging [28] and that telomere dysfunction is key to developing cancer [27]. However, Figure 4 shows that the specific incidence of colon cancer decreases with age, after about age 84. Others have made similar observations [22]–[24]. These findings cast doubt on the assertion that cancer is caused by aging.

Several artifacts could bias the age-specific incidence data. These include decreased rates of screening, different access to medical care, and birth cohort effects.

Colorectal screening rates decrease with age after 60 years. The National Survey of Ambulatory Surgery quantified the rate of outpatient colonoscopies (over 90% of colonoscopies are performed as outpatients) in 1994, 1995, 1996 and 2006 [30]. (The survey was not performed in the years between 1996 and 2006.) The rates are shown in Table 1. Based on these estimates, colorectal screening rates in the elderly population (over 85) were probably about 40% of the rate of 50 to 64 year olds.

**Table 1:** The rate of colonoscopies per 100,000 people in the population, estimated by the National Survey of Ambulatory Surgeries.

Age	1996	2000
50–64	1200	5000
85+	1200	2000

The increase in diagnosed cancers due to screening can be estimated from the data. Guidelines suggest beginning screening at 50 years of age. The colon carcinoma age-specific incidence data shows a small, but noticeable, increase over the expected rate at 50 years of age. From this, we estimate the number of new cases of colon carcinoma due to screening at about 2 per 100,000, when about 5000 per 100,000 are screened. Based on these numbers, we estimate that if screening rates did not decrease with age, the specific incidence of colon carcinoma would increase by about 40 cases per 100,000 population at age 85. This is not a significant difference. For comparison, the 95% confidence intervals are 50 to 200 (per 100,000) wide.

Most forms of cancer screening do not prevent cancers, but rather provide early detection. This effect is visible in the colon carcinoma age-specific incidence data, 50 and 51 year olds have slightly more cancers than expected, while 52 and 53 have slightly less than expected.

Access to medical care has a larger effect than screening. Colon carcinoma incidence data shows a significant increase at 65 and 66 years of age in the US population. This coincides with the age at which universal medical care is provided in the US. Since universal access to medical care begins at age 65 and does not end, it is unlikely to affect the incidence rate for people 85 to 99 years of age.

The drop in incidence after age 85 is not due to birth cohort effects. The expected value, if 100% of the population were susceptible, for age 99 is about 850 per 100,000. The observed value is about 227 with a 95% confidence interval of (96 to 357). The observed value is about one quarter of the expected value, if 100% were susceptible to colorectal carcinoma. If these women, born in 1900–1910, had a significantly reduced propensity to develop colorectal carcinoma, then we should see a correspondingly small incidence in women of age 63 to 73 years old recorded in 1973 (the earliest SEER data available). No such effect is noticeable in the 1973 data.

### Interpretations of the data

Two interpretations of the observed decrease in the specific incidence with age have been proposed: a frailty hypothesis [9], [31] and an elderly hypothesis [19]. The first, the frailty hypothesis, is that this decrease indicates the existence of two subpopulations: one subpopulation with an innate propensity to develop colon carcinoma and a second subpopulation with immunity developed at birth or an early age. The second interpretation, the elderly hypothesis, postulates that some biological process begins at an advanced age, which leads to decreased cellular proliferation and hence decreased cancer rates.

Pompei and Wilson have proposed that the mechanism behind the elderly hypothesis is cellular senescence. They point out that an experiment [32] that genetically altered mice to increase cellular senescence resulted in premature aging but decreased cancers.

Different forms of the frailty hypothesis have been proposed. Trichopoulos has suggested that hormonally regulated cancers originate *in utero*. This would explain a number of curious observations about breast cancer including the dramatic difference in incidence found in Japan and the USA [33]. Barker has suggested that not only cancers, but also other adult diseases have fetal origins [34], [35]. Others have also suggested that some chronic diseases are influenced by exposure to environmental factors early in life [36], [37]. Diabetes [38], schizophrenia [39], and lung disease [40] might also find their origins in early life.

Several known mechanisms could be responsible for the existence of two sub populations required for the frailty hypothesis. These include germ line mutations, somatic mutations early in life, and/or epigenetic modifications.

Simple germ line mutations have been ruled out. During the 1990's, significant resources were devoted to the identification of germ line mutations for the most common forms of cancers. This effort led to the identification of BRCA1 [41]. Certain mutations in BRCA1 significantly increase the risk that a woman will develop breast cancer. However, these mutations are rare and less than 10% of breast cancers in the US population occur in women with these mutations. Despite searching for similar genes in colon cancer [42], none have been found with the significance of BRCA1. No recurrent mutations are responsible for the progression of colon cancer [43]. More recent genome wide association scans for susceptibillity loci in colorectal cancer (for instance [44]–[46], and a recent meta analysis of many similar studies [47]) have identified some loci that might have a small influence on the heritability of colon cancer. However, colon cancer is still plagued by the missing heritability problem [48], [49].

Somatic mutations acquired early in life (during development) could propagate to encompass entire tissues. Embryonic cells are actively proliferating and a somatic mutation acquired early during development will be found in many cells. Irradiation of a fetus is known to increase the incidence of childhood cancers [50] presumably through the acquisition of somatic mutations. Somatic mutations acquired during development are known to be responsible for retinoblastoma, a type of childhood cancer [51].

Epigenetic alterations play a key role in the carcinogenesis process [52]–[54]. Modification of histones are a key regulatory step in transcription [55] and DNA damage repair [56]. Specific histone modifications have been identified that are common features of human cancers [57], [58]. Several approaches to determining genome wide methylation exist, but these approaches have not yet been widely applied to cancer as much as DNA sequencing [59].

### Risk factors, behavior and environment

Risk factors are often misunderstood as causes. Modifiable risk factors are alterable characteristics that increase the likelihood of a person developing a disease before dying. Factors that speed the development of the tumor will appear as significant changes in the risk factors for cancers that occur late in life (colon cancer), but will not appear as significant risk factors for tumors that occur earlier in life.

### Conclusion

In conclusion, our analysis shows that the age-specific incidence data for testicular and colon cancers is similar. This conclusion suggests that the etiology of colon carcinoma and testicular cancers might be similar. Testicular cancers are thought to originate *in utero*, colon cancers might also originate at an early age.

## Materials and Methods

Cancer registries count diagnosed tumors within a geographic area. The Surveillance, Epidemiology, and End Results (SEER) program of the National Cancer Institute (NCI) is considered the gold-standard for data quality for cancer registries. It now collects information on cancer cases from eighteen different geographic areas of the United States encompassing about 28% of the population of the United States.

Cancer cases are encoded with standardized histology coding descriptions. One example of a standardized coding system is the Collaborative Stage Schema.

We obtained the number of colon carcinoma cases diagnosed as a function of age from the SEER case files. We counted all colon carcinomas (encoded with the Collaborative Stage (CS) Schema v0202 equal to 18) that occurred in females in the year 2000. This amounted to 26119 cases. We chose only females because the age specific incidence is slightly different between men and women, and the population of women older than 90 years of age is much larger than the population of men older than 90 years of age.

Although the SEER case files contain all primary tumors recorded in patients of any age, the SEER population numbers range only from 0 to 84 years of age. All people older than 84 years of age are included into a single category labeled 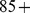.

We used the 2000 US Census to obtain an estimate of the 2000 SEER population for single years of age from 85 to 99. Specifically, we obtained the US national population as a function of age for women from the 2000 US Census Table PCT12, Sex by Age of Total Population, in Summary File 1 (SF1) 100 percent data.

The SEER population is not a random subsection of the United States population. The SEER geographic regions are chosen to ensure good representation of minority populations. Thus they are, relative to the US, overrepresented in certain minority populations. Because of this difference, the SEER population age distribution is slightly different from the age distribution of the entire nation.

We scaled the US population data to best match the SEER-17 population data from ages 60 to 84. We found the best match occurred when we multiplied the US population by a factor of 0.240, see Figure 1. This resulted in a median absolute error of 1.2% over the age ranges 60 to 84, and a median absolute error of 9% over the ages 0 to 59. Based upon this, we estimated the error in the population measurements for ages 85 to 99 at 10%.

Finally, we obtained the colon carcinoma age-specific incidence by dividing the number of cases, as a function of age, recorded in the SEER-17 case files by the scaled US population of women, as a function of age.

We obtained the age-specific incidence for testicular cancers directly from the SEER database. We selected, using SEER*Stat [60], all men who were diagnosed with testicular germ cell tumors classified as seminomas (Seminoma, in situ; Seminoma, NOS; and Seminoma, anaplastic) during the years from 2000 to 2009. This data set included 12,147 men. The associated population and confidence intervals were calculated by SEER*Stat.

Error estimates are given as 95% confidence intervals. Errors were estimated by SEERstat using [61] for testicular carcinomas and colon carcinomas for ages less than 85. For ages greater than 85, we estimated the error, 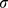, in the number, 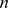, of diagnosed tumors as 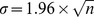. The error in the rate, (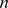/population)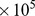, was estimated by adding in quadrature the error in the number of tumors, 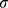, and the error in the population, which was estimated as 10% of the population number.

### The model

An extreme value model describes the statistical occurrence of the first (or last) event. Based on the observation that a tumor is diagnosed when the **first** cell accumulates a sufficient set of genetic alterations, we proposed [25] that the age-specific incidence data should follow a Weibull distribution. The probability density distribution, 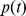, as a function of the age, 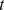, for a Weibull distribution is, 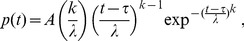
(1)for 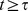. For 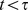, 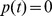. The Weibull distribution has four parameters: 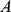 is a normalization factor, 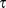 is a time shift, 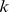 is known as the shape parameter, and 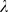 is called the scale parameter. The shape and scale parameters must be positive numbers. The parameter 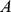 can be interpreted as the fraction of the population that would eventually develop the cancer, if everyone in the population lived forever.

The age-specific incidence is measured as a hazard function. Our derivation of the model involves a probability density distribution. The hazard function is equal to the probability density distribution divided by the survival function. Since carcinoma only occurs in a small fraction of the population, the hazard function and the probability density are nearly equal and the difference between the two is dwarfed by the sampling error from the SEER data. Therefore approximating the hazard function by the probability density function does not introduce a significant error.

To characterize how well the model fit the data, we determined the parameters that minimized the chi-square function, 
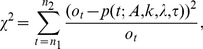
(2)which is the square of the difference between the observed values, 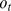 and the model's predictions, 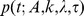 summed over all relevant ages. Each term of the sum is weighted by the inverse of the square of the error associated with the observed value. Since the counts are Poisson distributed, the estimated error is 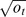. The relevant ages included any with at least 10 observed counts (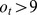). The minimization used the generalized reduced gradient algorithm of minimization [62]. We used multiple starting points for the parameters to ensure it does not converge on a relative minimum.

The optimal estimates of the parameters 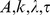 are those that minimize 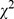, when comparing the hypothesized model 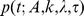 to each observed data point, 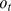.
